# Supplementary material for: Engineered Human Dendritic Cell Exosomes as Effective Delivery System for Immune Modulation
Source: Int J Mol Sci. 2023 Jul 11;24(14):11306. doi: 10.3390/ijms241411306 (PMC10379002; doi:10.3390/ijms241411306)
Supplement: Supplementary file 1 [file ijms-24-11306-s001.zip › ijms-2334285-supplementary.pdf]

Supplementary Material:

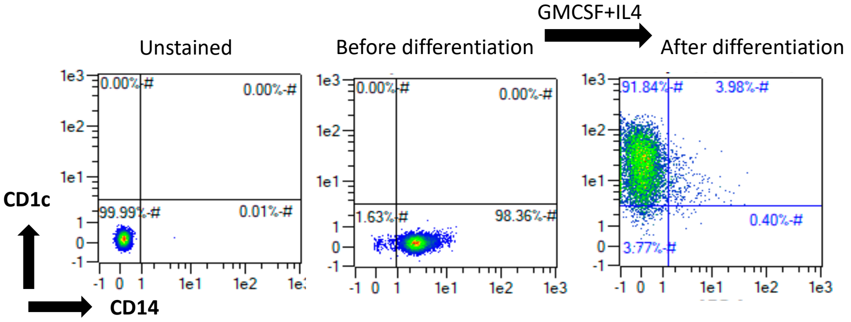

Figure S1: Purity of monocyte derived dendritic cells after differentiation

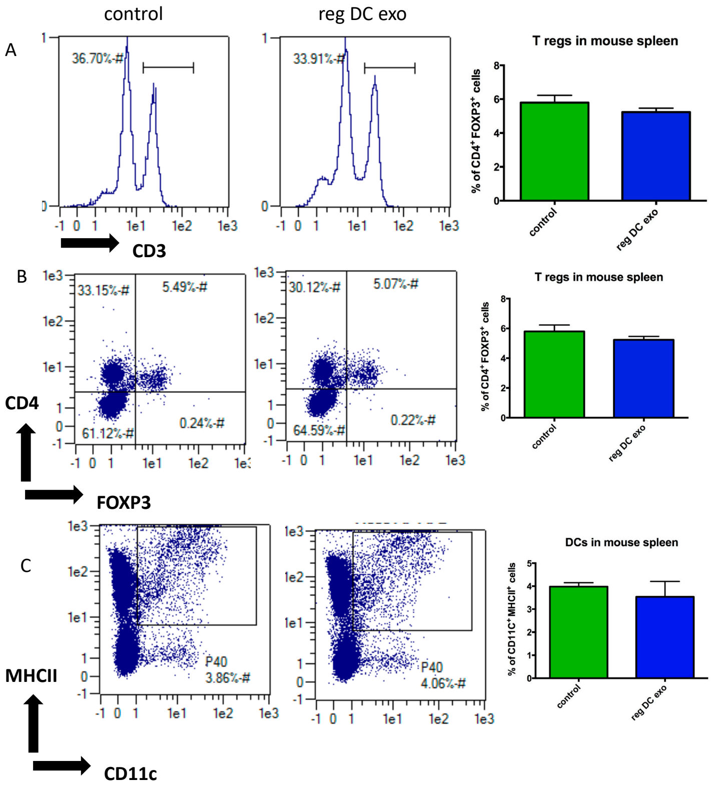

Figure S2: Immune cell populations in spleen of mice locally injected in the gingival tissue with regDCexo: A: flow cytometry scatter plot showing the % population of Tcells in spleen of mice treated with control PBS or regDCexo. B: flow cytometry scatter plot showing the % population of T regs (CD4+FOXP3+) in spleen of mice treated with control PBS or regDCexo. C: flow cytometry scatter plot showing the % population of DCs(CD11c+MHCII+) in spleen of mice treated with control PBS or regDCexo.

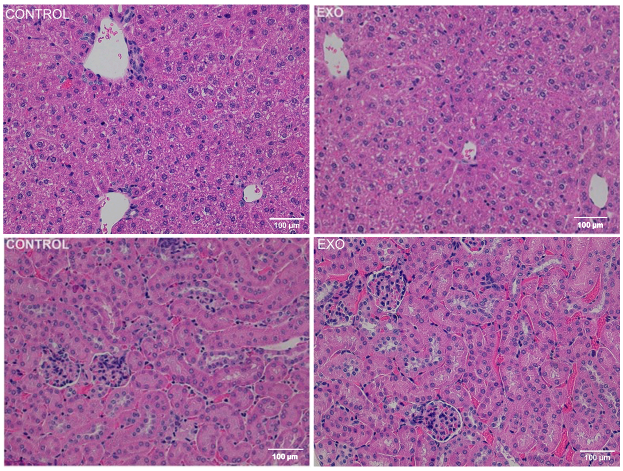

Figure S3: Histological micrographs showing liver tissues (upper panel) and kidney tissues (lower panel) of mice locally injected with PBS or regDCexo

Methodology for supplemental material:

Animals: For cytotoxicity and safety profiles of regDCexo, in our preclinical animal model, tissues were harvested from animals with ligature induced periodontitis and treated with regDCexo or PBS. A group received ligature plus local administration of 10 ul of PBS (control) (n=5), and a group received ligature plus local delivery of 10^8 particles of regDCs EXO suspended in 10 ul PBS (n=5). Kidney and liver from mice treated with regDCexo or PBS (control) were fixed in 4%PFA, paraffin embedded and then 5 microns sections were mounted on slides and stained with H&E. Analysis was performed blindly by a pathologist (Z.K.). Spleens were collected, and cells isolated and stained for flowcytometry analysis using the following antibodies: anti-mouse CD3 FITC; clone: 145-2C11 (Affymetrix, eBioscience, Thermofisher Scientific, Waltham MA, USA; Cat#: 50-112-9706), anti-mouse CD4 eFluor 450; clone: GK1.5 (Affymetrix, eBioscience, Thermofisher Scientific, Waltham MA, USA; Cat#: 48-0041-82), anti-mouse FOXP3 APC; clone: FJK-16s (Affymetrix, eBioscience, Thermofisher Scientific, Waltham MA, USA; Cat#: 17-5773-82), anti-mouse CD11c APC; clone: N418 (Affymetrix, eBioscience, Thermofisher Scientific, Waltham MA, USA; Cat#: 17-0114-82), anti-mouse MHCII viogreen; clone: M5/114 ((Miltenyi Biotech, Auburn, CA, USA; Cat#: 130-119-122).
